# Inhibition of EGFR, HER2, and HER3 signalling in patients with colorectal cancer wild-type for *BRAF, PIK3CA, KRAS*, and *NRAS* (FOCUS4-D): a phase 2–3 randomised trial

**DOI:** 10.1016/S2468-1253(17)30394-1

**Published:** 2017-12-16

**Authors:** Richard Adams, Ewan Brown, Louise Brown, Rachel Butler, Stephen Falk, David Fisher, Richard Kaplan, Phil Quirke, Susan Richman, Leslie Samuel, Jenny Seligmann, Matt Seymour, Kai Keen Shiu, Harpreet Wasan, Richard Wilson, Tim Maughan, FOCUS4 Trial Investigators

**Affiliations:** a Cardiff University and Velindre Cancer Centre, Cardiff, UK; b Edinburgh Cancer Centre NHS Lothian, Edinburgh, UK; c Medical Research Council (MRC) Clinical Trials Unit at University College London (UCL), London, UK; d All Wales Genetics Laboratory University Hospital of Wales, Cardiff, UK; e University Hospital Bristol NHS Foundation Trust, Bristol, UK; f Leeds Institute of Cancer and Pathology, University of Leeds, Leeds, UK; g Aberdeen Royal Infirmary, Aberdeen, UK; h University College London, London, UK; i Imperial College London, London, UK; j Centre for Cancer Research and Cell Biology, Queen's University Belfast, Belfast, UK; k Cancer Research UK/MRC Oxford Institute for Radiation Oncology, University of Oxford, Oxford, UK

## Abstract

**Background:**

A substantial change in trial methodology for solid tumours has taken place, in response to increased understanding of cancer biology. FOCUS4 is a phase 2–3 trial programme testing targeted agents in patients with advanced colorectal cancer in molecularly stratified cohorts. Here, we aimed to test the hypothesis that combined inhibition of EGFR, HER2, and HER3 signalling with the tyrosine kinase inhibitor AZD8931 will control growth of all wild-type tumours.

**Methods:**

In FOCUS4-D, we included patients from 18 hospitals in the UK with newly diagnosed advanced or metastatic colorectal cancer whose tumour was wild-type for *BRAF, PIK3CA, KRAS*, and *NRAS*. After 16 weeks of first-line therapy, patients with stable or responding tumours were randomised to oral AZD8931 (40 mg twice a day) or placebo. Randomisation was done by minimisation with a random element of 20%, minimisation by hospital site, site of primary tumour, WHO performance status, 16-week CT scan result, number of metastatic sites, and first-line chemotherapy regimen. The primary outcome was progression-free-survival. CT scans were assessed by local radiologists according to Response Evaluation Criteria in Solid Tumors (RECIST), version 1.1. Preplanned interim analyses were assessed per protocol and were agreed using multiarm multistage (MAMS) trial design methodology triggered by occurrence of progression-free survival events in the placebo group. The final analysis was assessed by intention to treat. This trial is registered at controlled-trials.com, ISRCTN 90061546.

**Findings:**

Between July 7, 2014, and March 7, 2016, 32 patients were randomised to study treatment, 16 to AZD8931 and 16 to placebo. At the first preplanned interim analysis (March, 2016), the independent data monitoring committee (IDMC) recommended closure of FOCUS4-D because of a lack of activity. At the final analysis (Aug 1, 2016), 31 patients had had a progression-free survival event (15 with AZD8931 and 16 with placebo). Median progression-free survival was 3·48 months (95% CI 1·51–5·09) in the placebo group and 2·96 months (1·94–5·62) in the AZD8931 group. No progression-free survival benefit of AZD8931 compared with placebo was noted (hazard ratio [HR] 1·10, 95% CI 0·47–3·57; p=0·95). The most common grade 3 adverse event in the AZD8931 group was skin rash (three [20%] of 15 patients with available data *vs* none of 16 patients in the placebo group), and in the placebo group it was diarrhoea (one [7%] *vs* one [6%]). No grade 4 adverse events were recorded and no treatment-related deaths were reported.

**Interpretation:**

The MAMS trial design for FOCUS4 has shown efficiency and effectiveness in trial outcome delivery, informing the decision to proceed or stop clinical evaluation of a targeted treatment within a molecularly defined cohort of patients. The overarching FOCUS4 trial is now aiming to open a replacement arm in the cohort with all wild-type tumours.

**Funding:**

Medical Research Council (MRC) and National Institute for Health Research (NIHR) Efficacy and Mechanism Evaluation programme, Cancer Research UK, NIHR Clinical Trials Research Network, Health and Care Research Wales, and AstraZeneca.

## Introduction

Signalling through HER receptors—ie, EGFR, HER2, HER3, and HER4—and their downstream pathways is a key mechanism that promotes proliferation and the malignant phenotype in cancer. EGFR has been recognised as a key pathogenic surface receptor in colorectal cancer for many years. A greater understanding of colorectal cancer biology followed by use of EGFR-targeted treatments has led to discovery of the importance of *BRAF, PIK3CA, KRAS*, and *NRAS* mutations in predicting a lack of response to EGFR-targeted therapy.^1^ The monoclonal antibodies cetuximab and panitumumab were developed to target EGFR on the surface of cancer cells. After licensing, cetuximab and panitumumab were reported to be ineffective in patients whose cancer expressed a somatic mutation in the *NRAS* or *KRAS* genes.^2^ Simple EGFR inhibition therefore has limitations, because both de-novo and acquired resistance can arise and result in lack of benefit for most of these biomarker-selected patients, thus driving interest in novel approaches to inhibit this pathway. However, EGFR remains a valid target in colorectal cancer within the population of patients wild-type for *KRAS, NRAS*, and *BRAF*, with proven clinical benefit.

**Research in context**

**Evidence before this study**

The HER family has been a focus of attention in colorectal cancer since the development and licensing of the EGFR monoclonal antibodies panitumumab and cetuximab. The mechanism of action of these agents remains to be elucidated fully but has led to preclinical research implicating other HER family members in de-novo or evolving resistance. Several molecules (both monoclonal antibodies and tyrosine kinase inhibitors) have been developed that aim to overcome resistance pathways in selected patients. The most successful of these approaches to date has been a combination of trastuzumab and lapatinib, which has shown efficacy in a small cohort of patients with metastatic colorectal cancer harbouring HER2-positive tumours. All other drugs that have been developed specifically to interact with the HER family have so far failed to show additional efficacy in colorectal cancer, above that evident for cetuximab or panitumumab. AZD8931 was designed to inhibit EGFR, HER2, and HER3 signalling. In two previous trials of AZD8931 as part of a combination strategy for breast cancer, no efficacy was recorded.

**Added value of this study**

Our study is the first randomised controlled trial to investigate use of AZD8931 in patients with colorectal cancer and in a prespecified molecular cohort of patients deemed most likely to be responsive to this approach. The multiarm multistage (MAMS) study design allowed optimisation of recruitment and early assessment of the drug in an efficient and effective manner.

**Implications of the available evidence**

AZD8931 showed no benefit in the first-line setting for metastatic colorectal cancer. The finding that this agent was ineffective in a molecularly defined subgroup of patients suggests that as monotherapy, AZD8931 has little to add in this disease type. The MAMS trial design has shown an ability to deliver an efficient and effective trial in molecularly defined cohorts of patients with metastatic colorectal cancer.

The proteins HER2 and HER3 (also known as ERBB2 and ERBB3, respectively) heterodimerise with EGFR and are mechanisms of resistance to EGFR inhibition. HER3 is a membrane-bound receptor protein that has extracellular heregulin and neuregulin binding domains but does not have an intracellular kinase domain, relying on heterodimerisation to other family members for downstream effects. HER3 expression is associated with poor prognosis in colorectal cancer.^3^ The protein has a central role in driving oncogenic signals in tumours,^4^ and preclinical and clinical data have led to the hypothesis that HER3 is an escape pathway to EGFR blockade through a compensatory shift to HER3 signalling, predominantly through the PI3K/AKT pathway.^5^, ^6^, ^7^ Moreover, clinical data indicate that HER3 overexpression predicts the lack of efficacy of panitumumab.^8^ HER2 is another member of the EGFR family of membrane-bound receptors. It is an orphan receptor, having no known associated ligands, but is activated through homodimerism and heterodimerisation with other EGFR family members, resulting in intracellular phosphorylation and cascaded downstream signalling. Growing in-vitro and in-vivo evidence suggests that HER2 might be overexpressed more frequently in patients with EGFR-dependent (all wild-type) tumours.^9^ Furthermore, this protein might be upregulated in acquired EGFR inhibitor resistance, and concomitant blockade of HER2 could increase efficacy and prolong activity in a synergistic fashion. Bertotti and colleagues^10^ developed a range of colorectal cancer xenograft models from genetically well-characterised, metastatic colorectal cancer samples. A cohort of these so-called xenopatients showed amplification of *HER2*, particularly in *RAS* wild-type tumours, suggesting enrichment in this cohort, which showed high and sustained sensitivity to combination EGFR and HER2 inhibition. Inhibition of EGFR, HER2, and HER3 signalling is postulated to reduce de-novo resistance, thereby increasing the proportion of patients showing benefit when compared with inhibition of EGFR only. Furthermore, this inhibition might slow the development of acquired resistance in patients with initially EGFR-dependent tumours, therefore providing greater clinical benefit than EGFR inhibition alone.

The convergence of the molecular understanding of colorectal cancer and clinical development of a wide range of targeted treatments demands evaluation of new agents within biologically defined subsets of patients whose tumours are most likely to benefit. Moreover, after the failure of many trials to show benefit for a new treatment in colorectal cancer, we clearly need a new paradigm to attempt to make progress. The idea of one research question for all patients is outdated in colorectal cancer, as it is with breast cancer, and is increasingly the case across all oncology.

We have developed a new approach to trial design that links novel treatment evaluation with concurrent assessment of biomarkers within a confirmatory phase 2–3 trial setting. Such a design will ultimately answer at least three research questions for several treatments and biomarkers. First, after a period of first-line chemotherapy, do targeted novel treatments provide signals of activity in different biomarker-defined populations, and second, do these targeted treatments improve outcomes definitively? Finally, is evidence of activity restricted to the biomarker-defined groups?^11^ Such a trial design fulfils the pressing need to deliver molecularly driven interventions to appropriately defined cohorts of patients in an efficient and effective fashion. The FOCUS4 programme of trials investigates such a design in patients with advanced colorectal cancer.

FOCUS4 was set up to provide a trial platform for rapid identification of patients whose tumours can be characterised either by the presence of specific mutations or by validated biomarkers that characterise biological cohorts. The trial uses an adaptive design that allows early preplanned interim analyses of molecular cohorts to review whether there is sufficient drug activity to justify continuing the trial being undertaken in that cohort.^11^ Clinical trials are costly, and prudent direction of resources towards drugs that look the most promising could improve the efficiency with which new drugs can be evaluated. In this Article, we report the findings of one of these molecular cohorts (FOCUS4-D), which reached its first interim analysis trigger point in March, 2016. The aim of FOCUS4-D was to test the efficacy of AZD8931 in patients with colorectal cancer whose tumours are wild-type for *BRAF, PIK3CA, KRAS*, and *NRAS* mutations.

AZD8931 is an orally active, equipotent tyrosine kinase inhibitor of EGFR, HER2, and HER3 signalling. In-vitro analysis of AZD8931 in ligand-driven cell assays shows potency exceeding that of gefitinib and lapatinib in this system for EGFR, HER2, and HER3 inhibition.^12^ In pharmacokinetic and dynamic analyses, a direct relation was recorded between the total amount of AZD8931 in plasma and inhibition of EGFR phosphorylation.^12^ Within FOCUS4-D, we excluded patients with PTEN loss or somatic *BRAF, PIK3CA, KRAS*, and *NRAS* mutations. The rationale for the exclusion of patients with *BRAF* mutations relates to two specific sets of data. First, *BRAF*-mutant colorectal cancer has significantly worse prognosis than does disease with wild-type *KRAS* and *NRAS*.^13^ Second, despite some conflicting evidence, *BRAF*-mutant tumours gain no additional benefit from targeted EGFR inhibition.^14^ Patients harbouring tumours with a *PIK3CA* mutation or PTEN loss were excluded specifically because data suggest the PI3K/AKT pathway is a resistance mechanism to EGFR inhibition.^15^

In the phase 2 Medical Research Council (MRC) COIN-B trial,^16^ patients who had metastatic *KRAS* wild-type tumours were treated with an intermittent strategy of oxaliplatin, intravenous fluorouracil, and folinic acid and randomised to either intermittent cetuximab (with intermittent chemotherapy) or continuous cetuximab (including single-agent maintenance through the interval between chemotherapy). These trial data showed that use of maintenance cetuximab in the interval was associated with an improvement in the duration of the progression-free interval from 3 months to 6 months.^16^ FOCUS4-D builds on these data to test if—in a more specific molecular cohort—AZD8931 will improve progression-free survival in the interval off chemotherapy.

## Methods

### Patients' registration and biomarker assessment

We undertook the FOCUS4-D trial at 18 hospital sites in the UK. Patients aged 18 years and older with newly diagnosed locally advanced or metastatic colorectal cancer were eligible for registration in the FOCUS4 trial programme either at the start of a 16-week regimen of first-line chemotherapy or up to 12 weeks into the regimen. Patients could not be registered in FOCUS4 unless a CT scan had been done within 4 weeks of starting chemotherapy (preregistration CT scan). CT scans were obtained at 8 weeks and 16 weeks after chemotherapy was started. A CT scan done at the end of the 16-week regimen was compared with the preregistration CT scan by local radiologists, using Response Evaluation Criteria in Solid Tumors (RECIST), version 1.1. Patients whose tumours had progressed were ineligible for randomisation and any further involvement with FOCUS4 ended at this time.

The 16-week duration of first-line chemotherapy was determined by reviewing data from three key trials: COIN,^17^ CAIRO3,^18^ and AIO0207.^19^ This period accounts for factors including maximum tumour response and tolerability by patients.

**Figure 1:**
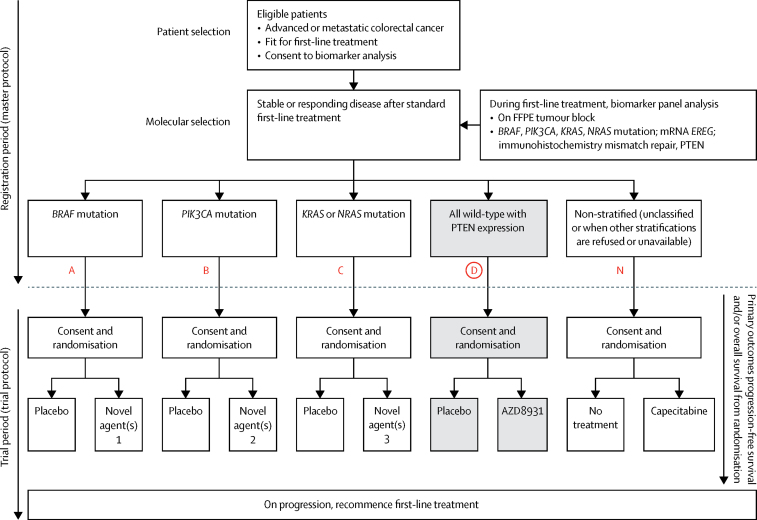
FOCUS4 trial programme schema Registration and randomisation processes and molecular stratification of patients in FOCUS4. Molecular cohorts are arranged in a hierarchy from left to right, such that a patient with both a *PIK3CA* and *KRAS* mutation will be classified in the *PIK3CA* mutation cohort. Red coloured letters indicate the FOCUS4 subtrials, with FOCUS4-D shaded grey. FFPE=formalin-fixed paraffin-embedded.

During the 16-week period of first-line chemotherapy, a sample of the patient's tumour was sent to one of two dedicated FOCUS4 biomarker laboratories in Leeds and Cardiff (UK) for assessment and stratification of the patient into one of four molecular groups. Figure 1 presents the molecular stratification hierarchy for the FOCUS4 trial programme. The technical components of the biomarkers and interlaboratory quality assurance have been described previously.^20^

**Figure 2:**
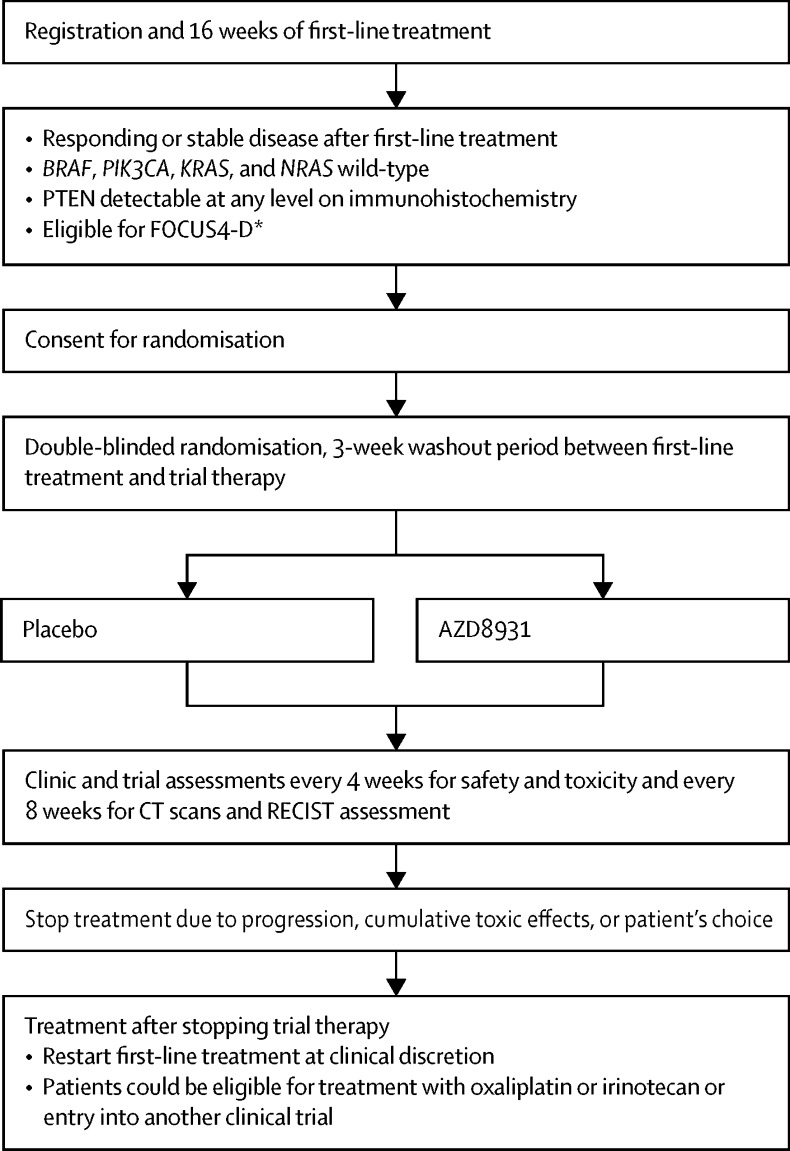
FOCUS4-D trial schema RECIST=Response Evaluation Criteria in Solid Tumors. *Criteria were age older than 17 years, no brain metastases, adequate organ function, WHO performance status 0–2, not pregnant, and CT scan within 4 weeks before randomisation.

Patients whose tumours had remained stable or responded to treatment according to CT were assessed for eligibility for FOCUS4-D. The biomarker assessment had to show wild-type status for *BRAF, PIK3CA, KRAS*, and *NRAS*, as well as PTEN expression on immunohistochemistry. Figure 2 presents the FOCUS4-D trial design with eligibility criteria. Full inclusion and exclusion criteria are available in the trial protocol, published on the FOCUS4 website. In addition to the eligibility criteria, patients had to have had a minimum 3-week gap between the last dose of chemotherapy or biological therapy and before the first dose of trial drug; a WHO performance status of 0–2; and adequate organ function, ascertained by an estimated creatinine clearance greater than 50 mL/min (according to local estimation method), serum bilirubin less than 1·5 × upper limit of normal (ULN), alanine aminotransferase, aspartate aminotransferase, and alkaline phosphatase less than 2·5 × ULN in the absence of liver metastases and less than 3·0 × ULN in presence of liver metastases, and a left-ventricular ejection fraction greater than 50% by multigated acquisition (MUGA) scan or echocardiography.

Ethics approval was granted by the National Research Ethics Service South Central Oxford—Panel C ethics committee (number 13/SC/0111). Regulatory approval was granted by the UK Medicines and Healthcare products Regulatory Agency (clinical trial authorisation [CTA] number 00316/0245/001-0001). All trial procedures and processes complied with the International Conference on Harmonisation's Good Clinical Practice guidelines. We asked patients to sign a consent form for registration and for analysis of the biomarker panel; additional written informed consent was obtained before randomisation in FOCUS4-D.

### Randomisation and masking

Randomisation was done by telephone to a centrally managed service at the MRC Clinical Trials Unit (CTU) at University College London (UCL). Patients were allocated to either AZD8931 or placebo by minimisation with a random element of 20% (both patients and clinicians were masked to treatment allocation). Minimisation factors were: treating hospital site, site of primary tumour (right colon, left colon, or rectum), WHO performance status (0, 1, or 2), 16-week CT scan result (stable disease, partial response, or complete response), number of metastatic sites (none, one, or two or more), and first-line chemotherapy regimen (fluorouracil, capecitabine, or neither; both oxaliplatin and irinotecan, oxaliplatin only, irinotecan only, or neither; and cetuximab or panitumumab, bevacizumab, or no monoclonal antibody). AZD8931 and placebo were identical in appearance, and were supplied by AstraZeneca (Cambridge, UK). Packaging, labelling, and distribution of AZD8931 and placebo were all undertaken by Fisher (Horsham, UK). Treatment was administered double-blind using an interactive web-based drug delivery system provided by Cenduit (Stirling, UK).

### Procedures

We asked patients to take oral AZD8931 (40 mg twice daily) or placebo until disease progression, death, or toxic effects. We also requested that patients complete a diary card on which the number of pills taken every day was recorded.

We assessed patients every 8 weeks by CT scan, which was reviewed at the treating hospital site according to RECIST, version 1.1. Measurements were collated centrally to ensure appropriate delineation of response and progression. Toxic effects and symptoms were assessed locally every 4 weeks from the start of trial treatment, using the National Cancer Institute's Common Terminology Criteria for Adverse Events (version 3.0). We followed up patients until progressive disease was identified on a CT scan, at which point we recommended the patient should restart first-line chemotherapy.

Treatment was stopped in the event of grade 3 or worse toxic effects or persistent toxicities judged medically significant or not tolerated by the patient, until the toxicity resolved to grade 1 or better. After stopping treatment (for a maximum of 28 days), trial therapy could be re-initiated at a reduced dose. For the first event, treatment was restarted at 40 mg in the morning and 20 mg at night; for the second event, treatment was restarted at 20 mg twice a day. A third event resulted in treatment discontinuation. Any stoppage for 28 days or more was not permitted and the patient was discontinued from trial therapy.

### Outcomes

The primary outcome of FOCUS4-D was progression-free survival, defined as time from randomisation to either disease progression (according to RECIST criteria) or death from any cause. The trial could be extended to include overall survival as a secondary outcome if adequate drug activity was seen for progression-free survival in the early interim analyses. Additional secondary endpoints included safety, toxicity, and tumour response.

### Statistical analysis

FOCUS4 uses a multiarm multistage (MAMS) design that allows preplanned analyses to be done to inform a decision on whether to continue the trial.^21^ We used the *nstage* MAMS function in Stata, version 14.1, to calculate the operating characteristics for each molecular trial.^22^ For FOCUS4-D we anticipated a recruitment rate of nine patients per month if 100 sites were open. For the placebo group, we assumed a median progression-free survival of 4·6 months, based on a similar group of patients being followed up in the COIN trial.^17^ A target hazard ratio (HR) of 0·5 was sought for progression-free survival, with a randomisation ratio of 1:1. Table 1 summarises the operating characteristics for FOCUS4-D, with predicted timelines for the staged interim analyses. Power was maintained at 85% after correction for two interim analyses and one final analysis. Target recruitment was 174 patients, with the first interim analysis timed to occur when nine progression-free survival events had occurred in the control group. At every interim analysis, we compared the observed HR with the critical HR for that interim analysis (table 1). If the HR was above the critical value, trial closure could be considered on the grounds of an absence of sufficient drug activity.

**Table 1:** Operating characteristics for FOCUS4-D trial

	Stage 1*	**Stage 2**†	**Stage 3**‡
Outcome	Progression-free survival	Progression-free survival	Progression-free survival
One-sided α	0·5	0·2	0·025
Power (overall power maintained at 85%)	0·91	0·95	0·95
Target HR	0·5	··	0·5
Critical HR	1·00	0·81	0·70
Time required (months)	5·9	6·3	7·1
Cumulative time (months)	5·9	12·3	19·4
Cumulative events required in placebo group (total events required)	9 (15)	30 (50)	59 (101)
Total expected cumulative randomisations	54	110	174

HR=hazard ratio.

* Safety and lack of sufficient activity.

† Lack of sufficient activity.

‡ Efficacy.

When an interim analysis was triggered by the prespecified number of progression-free survival events in the control group, we cleaned and analysed data and presented the results at a closed and confidential meeting of the independent data monitoring committee (IDMC), who were not involved with conduct of the study. Recommendations to continue or close the study were made to the trial steering committee (TSC), who would recommend their decision. If a decision was made to close the study, the TSC allowed the trial management group (TMG) to see the data to ensure there were no objections to study closure. When all committees agreed to trial closure, we informed study sites, the pharmaceutical company (AstraZeneca), and the trial funders, and we permitted no further patients entry into the study.

We did all analyses according to a predefined statistical analysis plan that was agreed before any data inspection. The primary outcome was prespecified to be analysed by intention to treat (final analysis). We did interim analyses in the per-protocol sample, which we defined as patients who completed at least one cycle of trial treatment (≥28 days). Per-protocol analyses were done for sensitivity. We analysed data with Stata, version 14.1.

We censored patients according to the following criteria. For survival status, we censored patients on the date they were last known to be alive, either via collection of prescription from the drug delivery system or attendance at a follow-up visit or CT scan. For patients who died before any follow-up visit or CT scan, we used the date of death as the date of the event and assumed death without progression. For progression-free survival, we censored patients without progression on the date of the last CT scan showing no progression.

We used Kaplan-Meier curves to present survival data and Cox regression modelling to estimate HRs, which we adjusted for the stratification factors that were used to minimise patients into allocated groups. Because the numbers of patients and events were small, we adjusted using the method of inverse probability weighting,^23^ and using the bootstrap method to estimate CIs. We tested the proportional hazards assumption by regressing scaled Schoenfeld residuals against the log of time.^24^ If evidence showed significant violation, we did a sensitivity analysis using restricted mean survival analysis.^21^

The FOCUS4 trial programme is registered at controlled-trials.com, ISRCTN 90061546.

### Role of the funding source

The funder had no role in study design, data collection, data analysis, or writing of the report. We informed the funder of results before submission of this Article and offered the funder the opportunity to comment on the data interpretation. DF and LB had access to raw data. RA had final responsibility for the decision to submit for publication.

## Results

**Figure 3:**
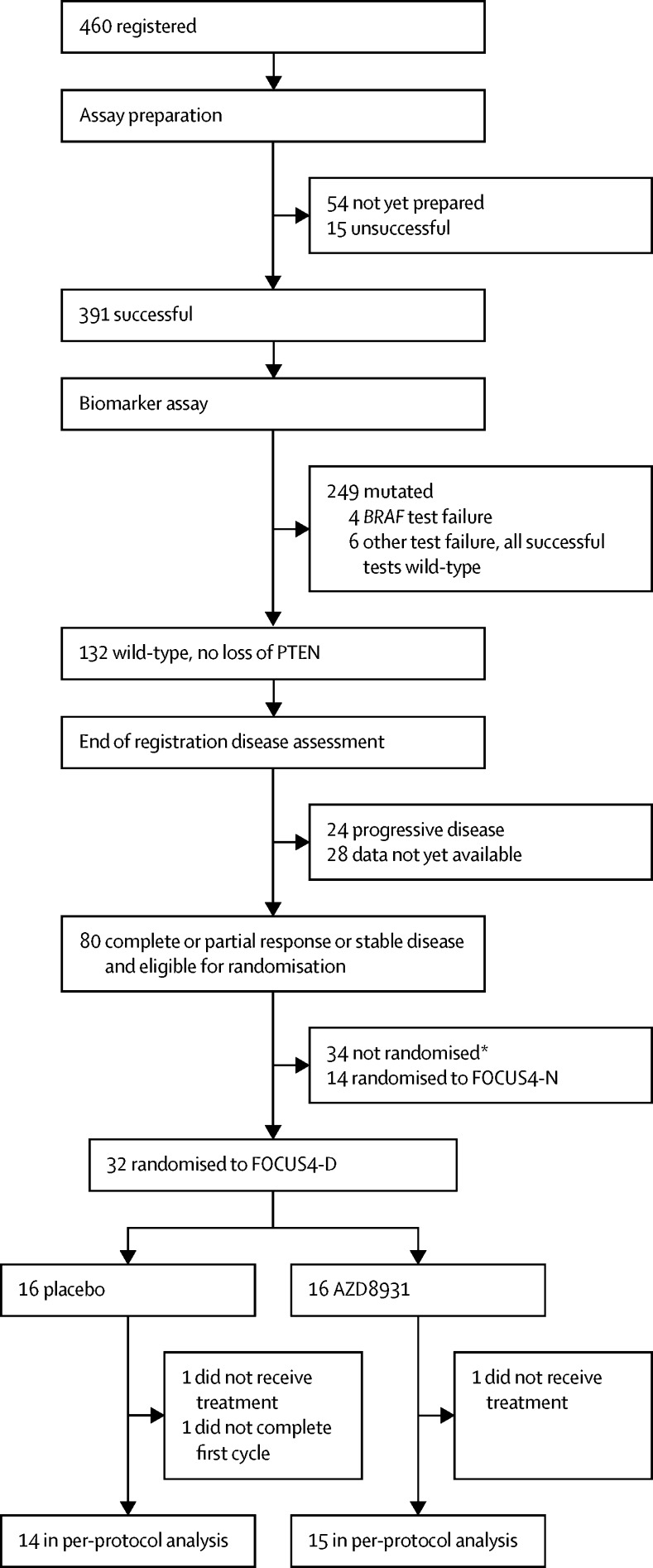
Trial profile *Reasons for non-randomisation not recorded.

**Figure 4:**
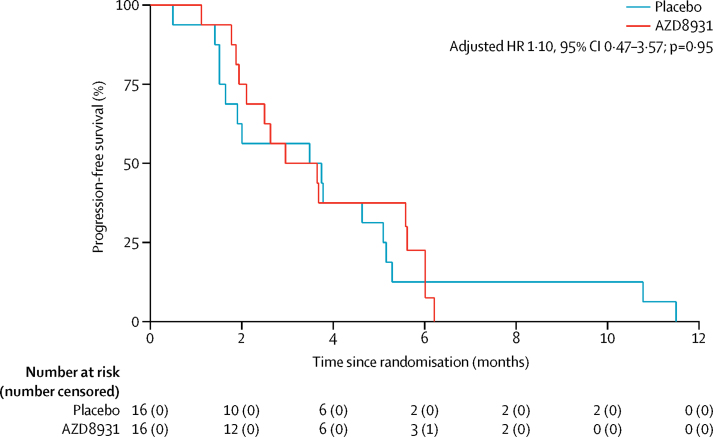
Progression-free survival (intention-to-treat analysis) HR=hazard ratio.

Between July 7, 2014, and March 7, 2016, 32 patients were randomised (16 into each treatment group) across 18 hospitals in the UK (figure 3). The groups were well balanced in terms of baseline characteristics (table 2). The first interim analysis was triggered in March, 2016, when nine progression-free survival events had occurred in the placebo group. The IDMC reviewed the data as part of a closed confidential meeting and made a recommendation to close the trial based on insufficient drug activity, because the observed HR did not fall below the critical HR threshold of 1·0, which was predefined as part of the multistage sample size calculations (table 1). This decision was subsequently endorsed by both the TSC and TMG. Trial recruitment was closed, and during subsequent follow-up of patients already entered into the study, further progression-free survival events occurred, such that 31 of 32 patients had a progression-free survival event by the time of the final analysis on Aug 1, 2016. No patients were lost to follow-up. Median progression-free survival was 3·48 months (95% CI 1·51–5·09) with placebo and 2·96 months (1·94–5·62) with AZD8931 (adjusted HR 1·10, 95% CI 0·47–3·57; p=0·95; figure 4).

**Table 2:** Baseline characteristics

		**Placebo (n=16)**	**AZD8931 (n=16)**
Age (years)		64 (58–74)	65 (57–71)
Sex			
	Male	13 (81%)	13 (81%)
	Female	3 (19%)	3 (19%)
Current state of primary tumour			
	Resected primary	11 (69%)	8 (50%)
	Unresected primary	5 (31%)	7 (44%)
	Unresected local recurrence	0	1 (6%)
Timing of metastases			
	Metachronous	4 (25%)	4 (25%)
	Synchronous	12 (75%)	11 (69%)
	Missing data	0	1 (6%)
Site of primary of tumour			
	Right colon	5 (31%)	5 (31%)
	Left colon	6 (38%)	6 (38%)
	Rectum	5 (31%)	5 (31%)
WHO performance status			
	0	12 (75%)	11 (69%)
	1 or 2	4 (25%)	5 (31%)
Disease assessment at end of first-line treatment			
	Partial response	8 (50%)	7 (44%)
	Stable disease	8 (50%)	9 (56%)
Number of metastatic sites			
	One	6 (38%)	9 (56%)
	Two or more	10 (63%)	7 (44%)
Fluoropyrimidine drug used during first-line treatment			
	Fluorouracil	11 (69%)	10 (63%)
	Capecitabine	5 (33%)	6 (38%)
Oxaliplatin or irinotecan used during first-line treatment			
	Oxaliplatin only	6 (38%)	6 (38%)
	Irinotecan only	9 (56%)	9 (56%)
	Neither	1 (6%)	1 (6%)
Monoclonal antibody used during first-line treatment			
	Cetuximab or panitumumab	4 (25%)	3 (19%)
	Bevacizumab	1 (6%)	1 (6%)
	No antibody	11 (69%)	12 (75%)

Data are number of patients (%) or median (IQR).

Two patients did not receive any treatment (one in each group), and one further patient in the placebo group did not complete their first cycle. Therefore, 29 patients were included in the per-protocol sample (14 in the placebo group and 15 in the AZD8931 group). The Cox regression analysis within the per-protocol sample (for sensitivity) generated an adjusted HR of 1·15 (95% CI 0·49–4·75; p=0·92) in favour of placebo. No statistical evidence was found that the proportional hazards assumption had been violated (Grambsch-Therneau test on log time scale, p=0·25 for intention-to-treat analysis and p=0·35 for per-protocol analysis), but because of the small sample size, a sensitivity analysis using restricted mean survival time analysis at 4 months was done. However, this test did not alter the results for either the intention-to-treat analysis (adjusted restricted mean 2·90 months [95% CI 2·34–3·46] in the placebo group *vs* 2·95 months [2·37–3·53] in the AZD8931 group; p=0·90 for difference) or the per-protocol analysis (3·08 months [2·61–3·56] *vs* 3·05 months [2·48–3·63]; p=0·94 for difference).

Among the 32 randomised patients, 11 (34%) received first-line irinotecan and fluorouracil. After progression, only four (36%) of these patients returned to their first-line therapy. Reasons for not doing so were the clinician's decision because of toxicity or disease progression. A further eight (25%) patients received first-line oxaliplatin plus capecitabine, and four (50%) of these patients returned to this regimen after progression. Reasons for not returning to first-line therapy were clinician's decision (toxicity [n=1], progression [n=1], and reported asymptomatic [n=1]) and entering another trial (n=1). A further seven (22%) patients received irinotecan and fluorouracil plus cetuximab or panitumumab as first-line therapy, and only one (14%) patient returned to their first-line regimen after disease progression. The reasons for not returning to first-line therapy were patient's choice (n=4), patient considered for resection (n=1), and missing data (n=1). Other first-line therapies included oxaliplatin and capecitabine plus bevacizumab (n=2), oxaliplatin and fluorouracil (n=2), capecitabine alone (n=1), and fluorouracil alone (n=1). 11 (34%) of 32 patients restarted their first-line therapy at the time of data collection. No patterns were noted in the reasons given for not restarting therapy. As of November, 2017, not all data for post-progression treatments were available.

Five of 32 patients needed dose reductions: one in the placebo group and four in the AZD8931 group. The patient in the placebo group had their evening dose reduced from 40 mg to 20 mg on their second cycle because of diarrhoea (grade 3). The dose reductions in the AZD8931 group were all evening dose reductions from 40 mg to 20 mg on the second or third cycles and were because of skin rash (grade 2 [n=1] and grade 3 [n=3]). One patient in the AZD8931 group subsequently had a morning dose reduction from 40 mg to 20 mg on their fourth cycle, also because of skin rash (grade 3), and another subsequently had a morning dose reduction from 40 mg to 20 mg on their sixth cycle, for dry eyes (grade 3). All dose reductions were maintained within the currently available data. Only one patient explicitly gave the reason for stopping trial treatment as toxicity. This patient was assigned AZD8931 and reported grade 3 dry eyes, grade 2 skin ulceration, and grade 2 skin rash. No serious adverse events were reported for this patient. Overall, few toxic effects were reported. Adverse events of grade 1–2 are listed in the appendix. Skin rash was the most frequent grade 3 adverse event, recorded in three (20%) of 15 patients in the AZD8931 group versus no patients in the placebo group; diarrhoea was the most frequent grade 3 adverse event in the placebo group, recorded in one patient (6%) versus one patient (7%) in the AZD8931 group (table 3). No grade 4 adverse events were reported. Five serious adverse events needed admission to hospital, four in the AZD8931 group (epigastric pain [grade 3]; back pain [grade 3], hyperbilirubinaemia [grade 4], and device-related infection [grade 2]; duodenal ulcer [grade 3]; and dehydration [grade 3, but subsequently fatal]) and one in the placebo group (chest infection [grade 3]). No treatment-related deaths were reported. Patients' compliance was 85% in the placebo group and 75% in the AZD8931 group.

**Table 3:** Adverse events of grade 3 or worse

	**Placebo (n=16)**	AZD8931 (n=15)*
Nausea	0	0
Vomiting	0	0
Diarrhoea	1 (6%)	1 (7%)
Stomatitis	0	0
Dry skin	0	0
Skin rash	0	3 (20%)
Acne	0	0
PPE	0	0
Anaemia	0	1 (7%)
Neutropenia	0	0
Thrombocytopenia	0	0
Hyperbilirubinaemia	0	1 (7%)
Elevated ALT or AST	0	0
Hypomagnesaemia	0	0
Cardiac toxicity	0	0
Pneumonitis	0	0
Infection	0	1 (7%)
Dry eyes	0	1 (7%)
Photophobia	0	0
Blurred vision	0	0
Conjunctivitis	0	0
Corneal ulcer	0	0
Fatigue	0	0
Paronychia	0	0
Epistaxis	0	0
Cystitis	0	0

Data are number of patients (%). Adverse events were graded according to the National Cancer Institute's Common Terminology Criteria for Adverse Events, version 3.0. No grade 4 or 5 events were reported. Adverse events of grade 1–2 are listed in the appendix. ALT=alanine aminotransferase. AST=aspartate aminotransferase. PPE=palmar-plantar erythrodysaesthesia.

* Data missing for one patient.

## Discussion

The FOCUS4 programme has delivered its first result in a molecularly selected cohort of patients with advanced colorectal cancer. In FOCUS4-D, AZD8931 failed to pass the first stage of assessment within the MAMS trial design—ie, the predefined critical hazard ratio of 1·0 at stage 1 (lack of sufficient activity; table 1) was not reached. However, this trial has set a new paradigm in molecularly stratified trials in colorectal cancer.

Two phase 2, randomised, combination trials of AZD8931 have been done previously, both in patients with breast cancer. The MINT study^25^ was stopped because of lack of efficacy (NCT01151215). In this trial, AZD8931 (40 mg or 20 mg twice daily) was compared with placebo in combination with anastrozole in postmenopausal women with hormone receptor-positive locally advanced or metastatic breast cancer who had never had endocrine treatment. The second randomised trial (THYME) did not meet its primary objective of prolonged progression-free survival when AZD8931 was added to weekly paclitaxel in patients with advanced breast cancer and low expression of HER2 (NCT00900627). The primary hypothesis in the THYME study was that low HER2 expression was a driver for heterodimerisation, which would be inhibited by AZD8931. In FOCUS4-D, we were unable to investigate subgroups effectively because of the small sample size. Specific areas of interest include the effect of anti-EGFR agents in the chemotherapy induction phase and pre-randomisation (six patients in total) and the role of primary tumour location as a marker of response to treatment. The findings of these three trials indicate a need for greater understanding of EGFR family heterodimers and their dynamic interaction with targeted agents. To date, heterodimers have been poorly assessed in vitro and in vivo because of a paucity of effective methods, which might have hampered more complete scrutiny of therapeutic agents such as AZD8931.

FOCUS4-D is the first of the FOCUS4 molecular cohorts to reach its first MAMS-defined interim analysis. The observed HR at the interim analysis was above the critical threshold for continuation of the trial, and the decision to close the trial was clearcut and unanimous across all oversight committees. We see this outcome as a success of the trial design, in that a decision to continue might have wasted clinical trial resources on recruiting and following up a further 142 patients, and closing the trial prevented other patients from being given an ineffective but potentially harmful treatment. FOCUS4-D was designed to detect a target HR of 0·5. Therefore, the probability of a final analysis of 174 patients showing an HR of 0·5 when the noted HR at this interim analysis was 1·10 is exceptionally small.

A limitation of the FOCUS4-D study was that some deviation took place from the assumptions that we made in our sample size calculations. The final sample size is small, with recruitment of only 32 of the maximum expected target of 174 patients. The median progression-free survival in the placebo group was 3·5 months, which is lower than we had anticipated in our sample size (4·6 months), and the higher event rate brought forward the trigger point for analysis (nine events in the placebo group). Owing to delays in site-opening of this complex trial, recruitment was also slower than anticipated, so this trigger occurred after randomisation of fewer patients than we had expected (32 instead of 54). However, since all but one patient had had a progression-free survival event by the time of the final analysis, we are reassured that further follow-up would not alter the conclusions of the study, and there is little reason to suspect that a further 22 patients (which would take our total sample size to the expected 54 patients at this analysis) would have responded any differently to the 32 patients reported here. Additionally, the fewer patients in the stage 1 analysis of lack of sufficient activity might increase any effect on outcome between the two treatment groups posed by trial imbalances in prognostic factors and choice of first-line treatment. For instance, the trial size makes it impossible to assess with any meaningful interpretation the effects of previous first-line treatment by EGFR inhibition with cetuximab or panitumumab (four patients in the placebo group and three patients in the AZD8931 group).

FOCUS4-D has shown no evidence of efficacy of single-agent EGFR, HER2, and HER3 inhibition with AZD8931 in patients with advanced colorectal cancer whose tumours are wild-type for *BRAF, PIK3CA, KRAS*, and *NRAS* after first-line induction therapy. Toxic effects were low, apart from skin rash in about 20% of patients. Early planned interim analyses with prespecified efficacy thresholds for lack of sufficient drug activity might be helpful in closing trials that are very unlikely to show benefit, which could facilitate more efficient use of clinical trial resources. New agents are currently being investigated for testing in this cohort of patients with all wild-type tumours.
