# Supplementary material for: Inhibition of EGFR, HER2, and HER3 signalling in patients with colorectal cancer wild-type for BRAF, PIK3CA, KRAS, and NRAS (FOCUS4-D): a phase 2–3 randomised trial
Source: Lancet Gastroenterol Hepatol. 2017 Dec 16;3(3):162–71. doi: 10.1016/S2468-1253(17)30394-1 (PMC6125825; doi:10.1016/S2468-1253(17)30394-1)
Supplement: Supplementary appendix [file mmc1.pdf]

THE LANCET
Gastroenterology & Hepatology

Supplementary appendix

This appendix formed part of the original submission and has been peer reviewed. We post it as supplied by the authors.

Supplement to: Adams R, Brown E, Brown L,et al, on behalf of the FOCUS4 Trial Investigators. Inhibition of EGFR, HER2, and HER3 signalling in patients with colorectal cancer wild-type for *BRAF*, *PIK3CA*, *KRAS*, and *NRAS* (FOCUS4-D): a phase 2–3 randomised trial. *Lancet Gastroenterol Hepatol* 2017; published online Dec 15. http://dx.doi.org/10.1016/S2468-1253(17)30394-1.

Table: Toxicities by randomised group, according to NCI CTC version 3.0

	Placebo		AZD8931	
	**G1/2**	**G3**	**G1/2**	**G3**
Nausea	4 (25%)	0 (0%)	4 (27%)	0 (0%)
Vomiting	1 (6%)	0 (0%)	4 (27%)	0 (0%)
**Diarrhoea**	**4 (25%)**	**1 (6%)**	**11 (73%)**	**1 (7%)**
Stomatitis	0 (0%)	0 (0%)	4 (27%)	0 (0%)
Dry skin	2 (13%)	0 (0%)	9 (60%)	0 (0%)
**Skin rash**	**3 (19%)**	**0 (0%)**	**11 (73%)**	**3 (20%)**
Acne	4 (25%)	0 (0%)	7 (47%)	0 (0%)
PPE	2 (13%)	0 (0%)	4 (27%)	0 (0%)
**Anaemia**	**6 (38%)**	**0 (0%)**	**6 (40%)**	**1 (7%)**
Neutropenia	2 (13%)	0 (0%)	2 (13%)	0 (0%)
Thrombocytopenia	3 (19%)	0 (0%)	1 (7%)	0 (0%)
**Hyperbilirubinaemia**	**1 (6%)**	**0 (0%)**	**3 (20%)**	**1 (7%)**
Transaminitis	0 (0%)	0 (0%)	4 (27%)	0 (0%)
Hypomagnesaemia	1 (6%)	0 (0%)	2 (13%)	0 (0%)
Cardiac toxicity	0 (0%)	0 (0%)	0 (0%)	0 (0%)
Pneumonitis	0 (0%)	0 (0%)	1 (7%)	0 (0%)
**Infection**	**2 (13%)**	**0 (0%)**	**3 (20%)**	**1 (7%)**
**Dry eyes**	**1 (6%)**	**0 (0%)**	**2 (13%)**	**1 (7%)**
Photophobia	1 (6%)	0 (0%)	1 (7%)	0 (0%)
Blurred vision	0 (0%)	0 (0%)	4 (27%)	0 (0%)
Conjunctivitis	0 (0%)	0 (0%)	1 (7%)	0 (0%)
Corneal ulcer	0 (0%)	0 (0%)	0 (0%)	0 (0%)
Fatigue	7 (44%)	0 (0%)	12 (80%)	0 (0%)
Paronychia	0 (0%)	0 (0%)	3 (20%)	0 (0%)
Epistaxis	0 (0%)	0 (0%)	1 (7%)	0 (0%)
Cystitis	0 (0%)	0 (0%)	1 (7%)	0 (0%)
**Total**	**16 (100%)**	**16 (100%)**	**15* (100%)**	**15* (100%)**

* missing data for one patient in AZD8931 arm

N.B. No toxicities of CTC grade 4 or 5 were reported.
